# Supplementary material for: Conditional chemoconnectomics (cCCTomics) as a strategy for efficient and conditional targeting of chemical transmission
Source: eLife. 2024 Apr 30;12:RP91927. doi: 10.7554/eLife.91927 (PMC11060718; doi:10.7554/eLife.91927)
Supplement: Figure 3—source data 3. [file elife-91927-fig3-data3.zip › Figure 3 source data3 part 1.zip!/Figure 3-Source data 3-part1/1071f3-1-CRISPResso_Report_jvi77nmx.zip!/CRISPResso_on_1071f3_4/9.Alleles_frequency_table_around_sgRNA_actactacatctccgtggag.pdf]

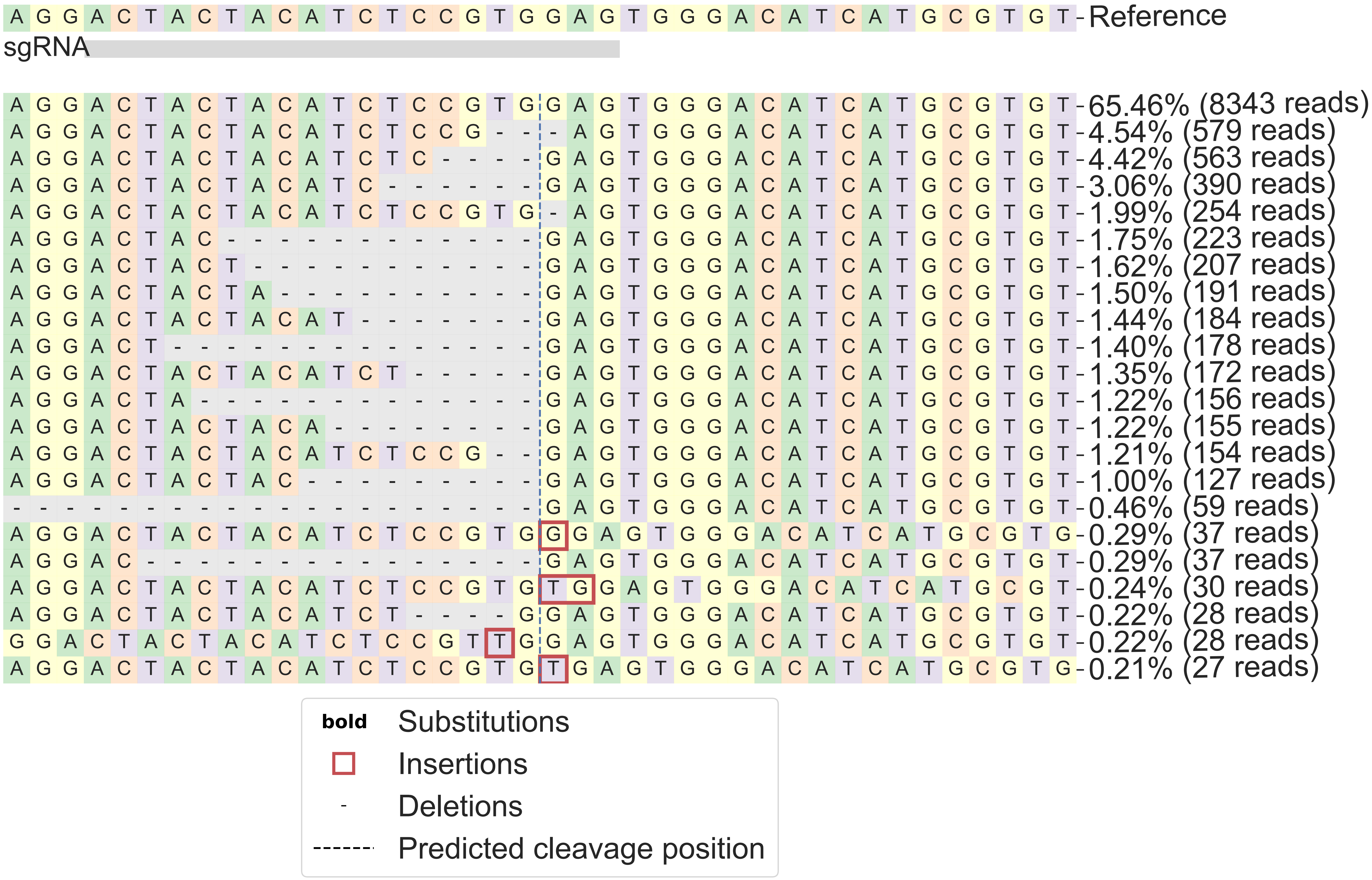

Reference
sgRNA
65.46% (8343 reads)
4.54% (579 reads)
4.42% (563 reads)
3.06% (390 reads)
1.99% (254 reads)
1.75% (223 reads)
1.62% (207 reads)
1.50% (191 reads)
1.44% (184 reads)
1.40% (178 reads)
1.35% (172 reads)
1.22% (156 reads)
1.22% (155 reads)
1.21% (154 reads)
1.00% (127 reads)
0.46% (59 reads)
0.29% (37 reads)
0.29% (37 reads)
0.24% (30 reads)
0.22% (28 reads)
0.22% (28 reads)
0.21% (27 reads)
bold Substitutions
Insertions
Deletions
Predicted cleavage position
